# Programming the Future: Epigenetics in the Context of DOHaD

**DOI:** 10.1289/ehp.125-A72

**Published:** 2017-03-31

**Authors:** Julie R. Barrett

**Affiliations:** Julia R. Barrett, MS, ELS, a Madison, WI–based science writer and editor, is a member of the National Association of Science Writers and the Board of Editors in the Life Sciences.

Recent studies have shown that variable responses to environmental exposures within a population arise in part from individuals’ genetic differences.^1^ ^,^ ^2^ ^,^ ^3^ Research on these differences is increasingly focusing on the epigenome, in which small chemical tags on DNA and associated proteins fine-tune genetic expression.^1^ ^,^ ^2^ ^,^ ^4^ A new review in *EHP* takes stock of the methods, analyses, and complexity of environmental epigenetics research in the context of the developmental origins of health and disease (DOHaD).^1^

**Figure:**
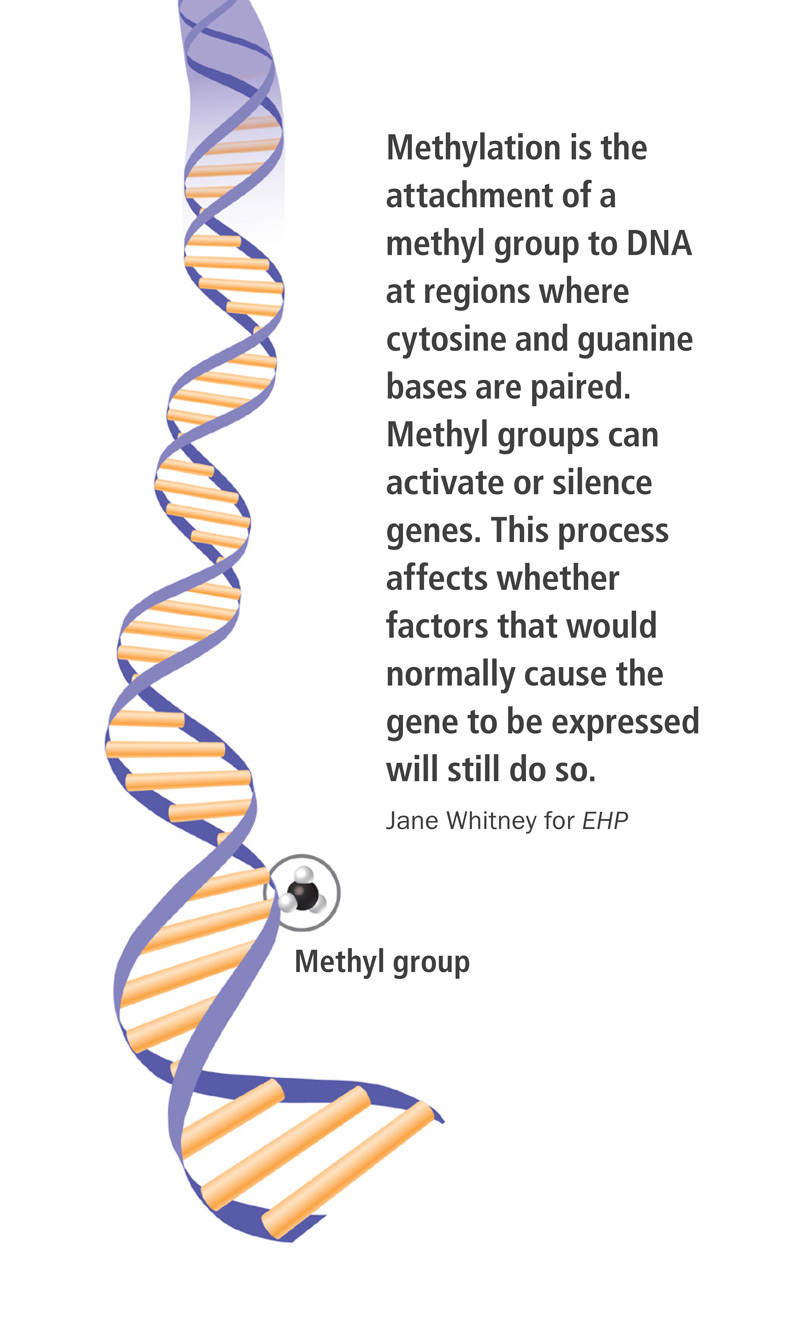
Methylation is the attachment of a methyl group to DNA at regions where cytosine and guanine bases are paired. Methyl groups can activate or silence genes. This process affects whether factors that would normally cause the gene to be expressed will still do so. Jane Whitney for EHP

According to the DOHaD hypothesis, environmental exposures during pre- and postnatal development can affect health years or even decades later.^1^ ^,^ ^4^ A potential bridge between these exposures and outcomes involves changes in, or reprogramming of, the epigenome.^2^ ^,^ ^3^ ^,^ ^4^ ^,^ ^5^ ^,^ ^6^

Epigenetic modifications are generally a critical part of normal development, helping to activate or silence specific genes during cell differentiation and thereby directing the formation of various tissues.^7^ But not all changes are benign, as suggested by associations between specific epigenetic alterations and disorders including cancer, neurodegeneration, and diseases of the cardiovascular and immune systems.^2^

Epigenetic programming has therefore drawn intense scrutiny as a potential biological mechanism through which environmental factors may influence health and susceptibility to disease.^2^ ^,^ ^3^ In addition, assessment of epigenetic alterations may help researchers to detect effects of exposures long after they have occurred and to better characterize disease risks.^1^ ^,^ ^2^ ^,^ ^6^

The review summarizes research pertaining to the detection and interpretation of epigenetic changes as the basis for the DOHaD hypothesis, which is a major objective of research under way in the federally funded Children’s Environmental Health and Disease Prevention Research Centers.^1^ “There are a lot of individuals within different children’s health centers who are very interested first and foremost in environmental health effects, but also in what role epigenetics can play in driving and explaining some of these health effects, specifically thinking about early-life development,” says first author Carrie Breton, an assistant professor of preventive medicine at the University of Southern California Keck School of Medicine.

For the review, Breton and her co-authors focused on DNA methylation, a specific type of epigenetic modification in which the presence or absence of a methyl tag can control whether a gene is active (transcribed) or silent (not transcribed). Researchers have identified subtle differences in blood measures of methylation between exposed and unexposed populations, with differences in methylation ranging from less than 2% up to 10%.^1^ “Often, we only see something like two to five methylated loci out of one hundred. That’s what, in this article, we are referring to when we talk about small magnitudes of change,” says Breton.

Whether such small changes are biologically meaningful is very much an open question in environmental health research. Previous genetic research has suggested that the location of epigenetic alterations can be a crucial determinant of the overall effect. For example, one study estimated that a 1% increase in methylation in a specific area of the *IGF2* gene halved transcription, while a 1% decrease doubled transcription.^8^ The scale of the estimated change in transcription is on par with that found for other genes in cancerous tissues.^1^ This finding illustrates that even small changes may have a large impact. In some cases, the effect may be indirect, with epigenetic changes poising a gene to react to a later trigger.^1^

However, it is important not to lose sight of the fact that epigenetic changes occur against the backdrop of the entire genome. “We can’t forget that it’s still a layer of information on top of our genetic code,” says Breton. She says that genome-wide association studies and many years of looking at genetic variation have shown that some diseases have an important genetic component.

“You really need to start thinking about how the epigenome responds to the environment and affects disease risk—but on top of the backbone of the genetic code,” Breton says. “You also need to think about measuring and adjusting for genetic variation or looking at interactions between the genetic code and the epigenetic code.”

Ultimately, researchers hope to be able to determine if specific epigenetic alterations bridge the gap between an environmental exposure and a particular outcome. “I think this is a very useful paper to a lot of people in terms of having a kind of common lexicon to work with [in terms of how to identify and describe small changes], and I like that they encourage discussion about small effect sizes,” says Daniele Fallin, a professor at the Johns Hopkins Bloomberg School of Public Health who was not involved in the review. “We are at that stage where there is still so much to learn; bringing people together and then communicating a common landscape are important.”

## References

[1] Breton CV Small magnitude effect sizes in epigenetic endpoints are important in children’s environmental health studies. Environ Health Perspect 125 4 511 526 2017, doi:10.1289/EHP595 28362264 PMC5382002

[2] Ladd-Acosta C Fallin MD The role of epigenetics in genetic and environmental epidemiology. Epigenomics 8 2 271 283 2016, doi:10.2217/epi.15.102 26505319

[3] Bakulski KM Fallin MD Epigenetic epidemiology: promises for public health research. Environ Mol Mutagen 55 3 171 183 2014, doi:10.1002/em.21850 24449392 PMC4011487

[4] Burris HH Baccarelli AA Environmental epigenetics: from novelty to scientific discipline. J Appl Toxicol 34 2 113 116 2014, doi:10.1002/jat.2904 23836446 PMC3867531

[5] Mitchell C DNA methylation, early life environment, and health outcomes. Pediatr Res 79 1–2 212 219 2016, doi:10.1038/pr.2015.193 26466079 PMC4798238

[6] Rozek LS Epigenetics: relevance and implications for public health. Annu Rev Public Health 35 105 122 2014, doi:10.1146/annurev-publhealth-032013-182513 24641556 PMC4480875

[7] Tang WW A unique gene regulatory network resets the human germline epigenome for development. Cell 161 6 1453 1467 2015, doi:10.1016/j.cell.2015.04.053 26046444 PMC4459712

[8] Murphy SK Gender-specific methylation differences in relation to prenatal exposure to cigarette smoke. Gene 494 1 36 43 2012, doi:10.1016/j.gene.2011.11.062 22202639 PMC3627389

